# Nasopharyngeal Pleomorphic Adenoma: A Rare Case Report and Review of the Literature

**DOI:** 10.1155/2018/2481370

**Published:** 2018-12-31

**Authors:** Serdal Celik, Osman Kilic, Tulay Zenginkinet, M. Tayyar Kalcioglu

**Affiliations:** ^1^Istanbul Medeniyet University, Faculty of Medicine, Department of Otorhinolaryngology, Goztepe Training and Research Hospital, Istanbul, Turkey; ^2^Istanbul Medeniyet University, Goztepe Training and Research Hospital, Department of Pathology, Istanbul, Turkey

## Abstract

Salivary gland tumors are rare among all head and neck tumors. Pleomorphic adenoma (PA) is the most commonly seen subtype, and 85% of the cases are located in the parotid gland. PA may very rarely be seen in minor salivary glands. Minor salivary gland PAs are mostly located in the hard and soft palates. Nasopharyngeal PA is very rare, and a total of 8 cases have been published to date. In this case report, a 51-year-old female patient who had nasopharyngeal PA with chondroid metaplasia is presented, and we review the relevant literature.

## 1. Introduction

Salivary gland tumors are rare and constitute less than 5% of all head and neck tumors [1]. Pleomorphic adenoma (PA), also known as benign mixed tumor, is the most common type among benign tumors of salivary glands. PA usually occurs in the 4th to 5th decades of life. It is located on the parotid gland, mainly on the superficial lobe, in 85% of cases. Even though PA rarely locates in the minor salivary glands, it is the most commonly seen minor salivary gland tumor and is most commonly located in hard and soft palates. These locations are followed by the upper lip in frequency [2–4]. PA originating from the nasopharynx is very rare, and only 8 cases have been reported in the literature to date [5–11]. In this case report, a 51-year-old female with nasopharyngeal PA is presented, and we review the relevant literature.

## 2. Case Presentation

**Figure 1:**
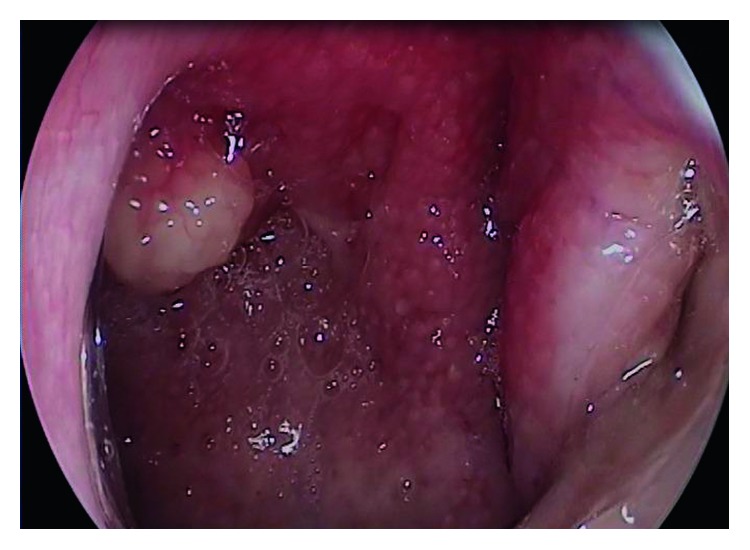
Preoperative nasopharyngeal endoscopy. The nasopharynx is seen in the posterior wall with a stalk-shaped mass and a 1 × 1 cm mass lesion.

**Figure 2:**
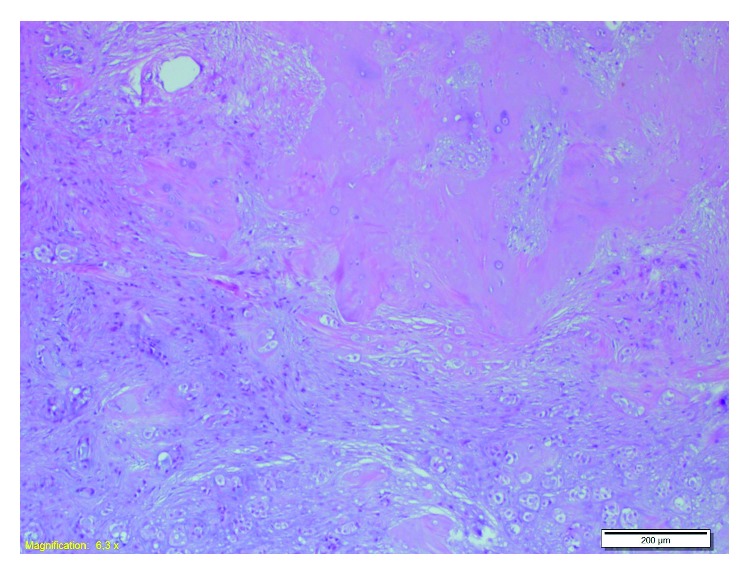
Pathologic specimen image. Pleomorphic adenoma showing chondroid metaplasia (10 H&E).

**Figure 3:**
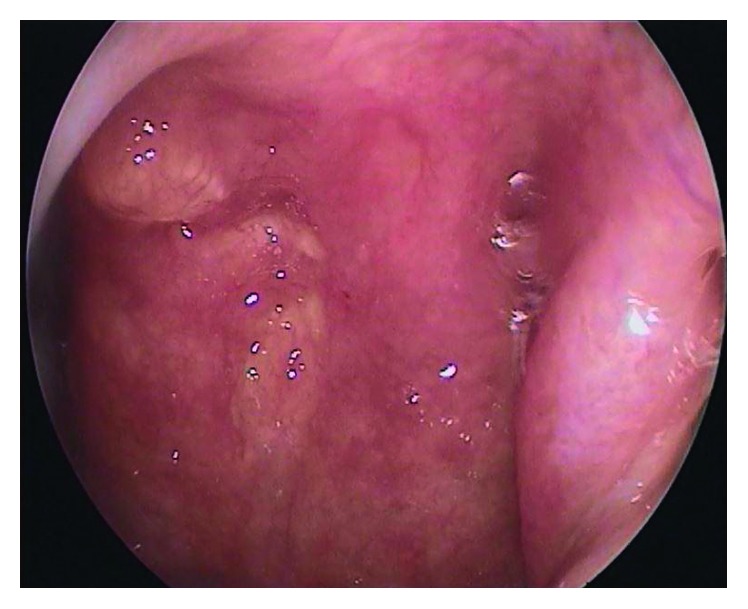
Postoperative 1st year image after punch biopsy. There was no significant difference between this and the appearance in the early postoperative period.

A 51-year-old female patient came to our clinic with postnasal drainage and intermittent nasal obstruction. Oropharynx examination was normal. There were no features on anterior rhinoscopic examination. On nasopharyngeal endoscopic examination, there was an approximately 1 × 1 cm mass with a smooth surface on the posterior wall of the nasopharynx (Figure 1). There were no other abnormal features on ENT examination. The nasopharyngeal mass was completely removed with the pedicle under local anesthesia. Pathologic evaluation of the specimen was reported as “pleomorphic adenoma showing chondroid metaplasia” (Figure 2). Complementary surgery was offered, but the patient did not accept additional intervention. Follow-up with endoscopic examination was performed every 3 months. No recurrence was seen during postoperative 1-year endoscopic follow-up (Figure 3). No additional masses were found in any head or neck region during the period of follow-up.

## 3. Discussion

When a nasopharyngeal mass is detected in an adult patient, nasopharyngeal malignancy is usually suspected. Most common types of nasopharyngeal cancer are keratinising squamous cell carcinoma, nonkeratinising carcinoma, and basaloid squamous cell carcinomas [12, 13]. Malignant diseases arising in the nasopharynx are asymptomatic in the early stage. They may be confused with other benign lesions. Therefore, diagnosis of nasopharyngeal malignancy is usually delayed [13]. Although the adenoid tissue usually shrinks in adolescence, it may be a cause of nasal masses in adults [12]. Benign lesions in the nasopharynx of adults also include cystic lesions such as branching cleft cyst, Tornwaldt cyst, and mucus retention cyst [14, 15].

PAs are the most common salivary gland tumors and comprise 45–70% of all salivary gland tumors [16, 17]. They are most commonly seen in the parotid gland and comprise 65% of all neoplasms originating from the parotid [17]. PAs, also known as benign mixed tumors, have various morphologic patterns and subtypes and comprise myoepithelial and epithelial cells. Macroscopically, a PA has a capsule. However, this is not a real capsule. The compressed normal salivary gland tissue takes the form of a false capsule. These tissues often show finger-like (pseudopod) extensions into the normal tissue [4]. The recurrence rate in cases of enucleation due to focal infiltrations of the tumor capsule and pseudopods of the pleomorphic adenoma is 20–45% [3]. No recurrence was observed during the 1-year follow-up period in our case.

PA is typically located in the parotid lower pole or the superficial lobe. Deep lobe localization is rare [3]. PA is usually a slow-growing asymptomatic mass, seen between the 4th and 5th decades of life [18]. The patients are usually females (64.5%). The current case was a 51-year-old female with characteristics similar to those described in the literature.

Nasopharyngeal PA is seen very rarely. According to the sources available to us, there have been only 8 cases reported in the literature [5–11]. Detailed demographic and clinical characteristics of these 8 cases are given in Table 1. PA cases with nasopharyngeal locations usually are referred to clinics because of nasal obstruction, nasal bleeding, dysphagia, and serous otitis-induced hearing loss due to Eustachian tube dysfunction. Serous otitis media (SOM) was detected in 5 of these 8 cases [5, 6, 9–11]. Some other pathologies such as adenoid vegetation or nasopharyngeal carcinoma can also cause SOM. SOM can also be seen in benign pathologies such as choanal polyps that can grow to cause obstruction at the entrance of the Eustachian tube [17]. All possible masses should be kept in mind if the patient has SOM. In the current case, because of the location and size of the tumor, it was clear that the patient did not have SOM.

Pleomorphic adenoma-related squamous cell carcinoma (SCC) has been detected in 3 of 8 cases published in the literature [5, 7]. In two of these cases, total surgical excision was performed by the authors, and no recurrence was observed during 20 months of follow-up [5]. In another case report by Furukawa et al., recurrence was not observed during 2 years of follow-up of a patient who underwent radiotherapy after total excision [7]. In order to be able to say something definite in this regard, a histopathological evaluation of the tissue is necessary. In the current case, the follow-up was only made with endoscopic examinations because the patient did not allow any additional interventions. According to our endoscopic examinations in the current case, neither malignant transformation nor recurrence was observed during 1 year of follow-up.

Surgical procedures such as lateral rhinotomy and transnasal endoscopic approaches used for the other nasopharyngeal benign tumors can be used for this mass [18]. In a case report published by Martínez-Capoccioni et al., excision of the posterior nasal septum was performed with a transnasal endoscopic approach, and the mass was totally excised with a better surgical view [9]. Surgically, total excision is the preferred method of reducing the risk of recurrence [19]. In our case, total excision of the mass with transnasal endoscopic approach was performed.

In conclusion, very rarely seen nasopharyngeal PAs should be kept in mind in the differential diagnosis of nasopharyngeal masses. The gold standard for treatment of PA is total surgical excision of the mass.

## Figures and Tables

**Table 1:** Nasopharyngeal pleomorphic adenoma cases in the literature.

	Age	Sex	Treatment	Follow-up duration (months)	Recurrence	Multiple location	Complaint	Size (cm)	Malignancy
Ryu et al. [5]	57	M	Surgery	12	No	No	Nasal obstruction	3.3	SCC
Ryu et al. [5]	37	M	Surgery	6	No	No	SOM	3.5	SCC
Lee et al. [6]	78	M	Surgery	20	No	No	Tinnitus, SOM	N/A	No
Furukawa et al. [7]	51	F	Surgery + RT	20	No	No	Nasal obstruction	N/A	SCC
Berrettini et al. [8]	51	F	Surgery	6	No	No	Nasal obstruction	4	No
Martínez-Capoccioni et al. [9]	52	F	Surgery	52	No	No	SOM	2	No
Maruyama et al. [11]	80	F	Surgery	24	No	No	SOM	2	No
Yazıcı et al. [10]	62	M	Surgery	12	No	No	SOM	2	No
Current case	51	F	Surgery	12	No	No	Nasal obstruction	1	No

SCC: squamous cell carcinoma; SOM: serous otitis media; RT: radiotherapy; M: male; F: female.
